# CD117^+^CD44^+^ Stem T Cells Develop in the Thymus and Potently Suppress T-cell Proliferation by Modulating the CTLA-4 Pathway

**DOI:** 10.1186/s13287-017-0495-4

**Published:** 2017-03-09

**Authors:** Yang Wei, Zhansheng Hu, Wen Gu, Gang Liu, Bingyin Shi, Enqi Liu, Tie Liu

**Affiliations:** 1 Immunology and Tumor Research Instituted, the First Affiliate Hospital, Xi’an Jiaotong University Health Science Center, Xi’an, Shaanxi 710061 People’s Republic of China; 2 Core Research Laboratory, the Second Affiliated Hospital, Xi’an Jiaotong University Health Science Center, Xi’an, Shaanxi 710049 China; 3 The First Affiliated Hospital, Jinzhou Medical University, Liaoning, 121004 People’s Republic of China; 4 Clinical Research Center, Guangdong Medical Collage, Zhanjiang, Guangdong 524001 China; 5 The School of Medicine, Xi’an Jiaotong University Health Science Center, Xi’an, Shaanxi 710061 China

**Keywords:** CD117, CTLA-4, CD25, CD44, Stem cell, Flow cytometry

## Abstract

**Background:**

CD117 is expressed on double-negative (DN; CD4^–^CD8^–^) cells (Nat Rev Immunol 14:529–545; 2014), but whether it is expressed in other stages and its subsequent functions are unclear. We used an improved method of flow cytometry to analyze different populations of thymocytes (Sci Rep 4:5781; 2014). The expression of CD117 and CTLA-4 were directly assayed in the early stage of thymocytes.

**Methods:**

Flow cytometry was used to analyze different populations of thymocytes, and T-cell proliferation assays, RT-PCR, and real-time RT-PCR were used to characterize the stem cells and examine the function of CD44^+^CD117^+^ cells.

**Results:**

In DN cells, CD117 expression was greatest on CD44^+^CD25^+^ cells (DN_2_), followed by CD44^+^CD25^–^ (DN_1_), CD44^–^CD25^+^ (DN_3_), and CD44^–^CD25^–^ (DN_4_) cells. In thymocytes, CD117 expression was highest in DN cells, followed by single-positive (SP; CD4 or CD8) and double-positive (DP; CD4^+^CD8^+^) cells.  Especially, CD117 expression was positively associated with CD44 and CTLA-4 expression. CTLA-4 expression was highest in DN cells, followed by SP and DP cells. CTLA-4 expression was positively associated with CD25, CD44, and Foxp3 expression. CD44^+^CD117^+^ T cells expressed more CTLA-4, which suppressed T-cell proliferation and blocked CTLA-4 to cause antibody-induced T-cell proliferation.

**Conclusion:**

These results suggest that CD44^+^CD117^+^ T cells are stem cells and a specific T-cell phenotype that initially develops in the thymus, but they do not progress through DN_3_ and DN_4_ stages, lack a DP stage, and potently suppress T-cell proliferation and modulate the CTLA-4 pathway.

## Background

T-cell development in mice begins with hematopoietic progenitor cells that migrate from the bone marrow to the thymus [1, 2]. The earliest precursors in the T-cell lineage are found within thymocyte populations and lack CD4 and CD8 expression, so they are referred to as CD4^−^CD8^−^ or double-negative (DN) thymocytes [3]. DN thymocytes consist of four subpopulations based on CD44 and CD25 expression [4]. DN cells that express CD4 and CD8 are CD4^+^CD8^+^ or double-positive (DP) thymocytes before differentiating into single-positive (SP; CD4^+^ or CD8^+^) thymocytes [5].

Stems cells include embryonic stem (ES) cells, induced pluripotent stem (iPS) cells, adult tissue stem/progenitor cells, and cancer stem cells. Mesenchymal stem cells (MSCs) have been shown to inhibit abnormally activated immune functions in dendritic cells, T cells, and B cells; however, preclinical efficacy studies to evaluate MSCs in autoimmune animal models have been contradictory [6–8].

Mammalian CD117 (c-*kit*) gene protein is a stem cell marker and a type III tyrosine kinase receptor [9, 10], widely expressed in hematopoietic stem cells (HSC), myeloid progenitor cells, as well as pro-B cells and pro-T cells [11–13]. In the hematopoietic system this protein contributes to cell growth, metabolism, proliferation, and survival [14, 15] during gametocyte development, pigmentation, and intestinal motility [16], and in the immune system contributes to inflammation [17]. The four DN subsets are defined by surface expression of c-*kit*, CD44, and CD25. Cells at the DN_3_ stage are positive for c-*kit* ^lo^CD44^−^CD25^+^. CD25 expression is downregulated as cells transition to the DN_4_ cell stage [14, 18].

Nanog is an important transcription factor for maintaining self-renewal and pluripotency of ES cells and regulates the fate of inner cell mass of blastocysts in the embryo during early development. The homeoprotein Nanog is required for maintenance of pluripotency in mouse epiblast and ES cells [19].

Cytotoxic T lymphocyte antigen-4 (CTLA-4) is an inhibitory relative of the T-cell costimulatory molecule, CD28. Whereas CD28 signaling promotes T-cell activation, CTLA-4 serves an immunoregulatory function by suppressing the T-cell response [20–22]. CTLA-4 also has intrinsic functions for T-conv cells and regulates trafficking of self-reactive T cells [23, 24].

Flow cytometry can be used to characterize single cells in an efficient and expedient manner [25], and can be used to describe DN cells which are present in limited amounts in the mouse thymus. Previously, a highly reproducible multi-color flow cytometry method was developed to increase thymocytes collected from 10^4^ to more than 10^6^ [26]. Using this method, we identified genes with low expression in DN cells and this permitted characterization of DN cell subsets.

In the present study, CD117, CTLA-4, CD4, CD8, CD25, and CD44 expression was measured in thymocytes and expression of CD117 and CTLA-4 was assayed directly in thymocytes and CD117^+^ stem T cells in the thymus and spleen. These data provide evidence of a relationship among CD117, CTLA-4, CD25, and CD44 cell surface markers on DN cells and other thymocytes.

## Methods

### Animals

Female C57BL/6 mice (Taconic Farms, Germantown, NY, USA) were maintained under specific pathogen-free conditions and were studied at 4–6 weeks of age, according to protocols approved by the Institutional Animal Care and Use Committee at Xi’an Jiaotong University, China.

### Flow cytometric analysis and cell sorting

Thymocytes and splenocytes were obtained from naive mice and suspended in PBS plus 1% fetal calf serum (FCS). To avoid nonspecific binding to mouse Fc-γ receptors, cells were blocked with mouse CD16/CD32 mAb (0.25 μg/100 μl; BD Biosciences, Franklin Lakes, NJ, USA) for 15 min at room temperature. Cells were washed and then stained with FITC anti-CD4 (clone RM4-5), PercP5.5-anti-CD8 (clone 53-6.7), APC-CY7-anti-CD44 (clone IM7), PE-CY7-anti-CD25 (clone PC61), PE-anti-CD117 (clone 2B8), FITC-CD117 (Clone 2B8), and APC-anti-CD3e (Clone 145-2C11) antibodies for 60 min at 4 °C. For some samples, surface-stained cells were fixed/permeabilized with a Cytofix/Cytoperm Kit (BD Biosciences) and stained with PE-anti-Foxp3 (clone FJK-16 s; eBioscience) and APC-anti-CTLA-4 (clone UC10-4B9; eBioscience) antibody for 45 min at 4 °C. The corresponding isotype controls (Armenian Hamster IgG, and Rat IgG2b,k) were purchased from eBioscience. Cells were sorted and analyzed using a FACSAria II, and data were analyzed using BD FACSDiva software (BD Biosciences).

### Culture medium and antibodies

RPMI 1640 medium containing 10% FCS was used for CD4 naive T-cell culture. Antibodies used for cell culture were anti-mouse CD3 (145-2C11) and anti-mouse IL2 (300 u/ml) (BD BioSciences, San Jose, CA, USA).

### Coculture system development

A coculture system was developed using a six-well Transwell plate (0.4 μm pore size insert; Corning Incorporated, Corning, NY, USA).

### Naive T cell and CD44^+^CD117^+^ cell sorting and stimulation

Lymphocytes from the spleen and thymus were stained with fluorochrome-conjugated antibodies, CD4 (FITC), CD8 (PerCp5.5), CD44 (APC-CY7), and CD117 (PE) for 45 min at 4 °C. Cells were then washed twice and resuspended at a density of 1 × 10^8^ cells/ml. Naive CD4, CD4^+^CD44^+^CD117^+^, and CD8^+^CD44^+^CD117^+^ cells were sorted using BD FACS Aria II. Anti-CD3 antibody was coated onto 24-well flat-bottom plates at 1 μg/ml in PBS overnight and then washed once with PBS. Naive T cells and CD4^+^CD44^+^CD117^+^ (CD8^+^CD44^+^CD117^+^) were then placed into the wells at a density of 1 × 10^6^ cells/ml in culture. Cells were incubated at 37 °C containing 5% CO_2_ for 7 days.

### T-cell proliferation assays

Cell proliferation was quantified using a Cell Trace CFSE Cell Proliferation Kit (Invitrogen, Carlsbad, CA, USA). Splenic T cells were prepared by passing splenocytes through nylon-wool columns (10^8^ splenocytes were incubated in the column for 1 h at 37 °C in 5% CO_2_ before eluting with RPMI medium), in which CD4^+^CD117^+^ cells (from flow cytometric cell sorting) were added to effecter cells (splenic T cells) at ratios of 1:20, 1:10, and 1:5. Cells were incubated with 2.5 μM CFSE (Invitrogen, USA) in PBS for 20 min at 37 °C. An excess of ice-cold RPMI 1640 with 10% FBS was added to the cells to quench the reaction. CFSE-labeled cells (1 × 10^6^/well) were washed twice with RPMI 1640 culture medium and then cultured in complete RPMI 1640 medium with 2 mM l-glutamine (Gibco), supplemented with 10 mM HEPES, 1% penicillin–streptomycin solution (Gibco), and 10% FBS (Gibco). A total 3 μg/ml of ConA or IL-2 and anti-CD3 antibody was added for polyclonal stimuli. Samples not stimulated with ConA or IL-2 and anti-CD3 were used as negative controls. Cells were then incubated for 7 days and stained with CD4 and CD8. T-cell proliferation was measured by quantifying CFSE fluorescence intensity via flow cytometry.

### CTLA-4 block assay

To block CTLA-4, 8 μl anti-mouse CD152 (CTLA-4) or isotype Ctrl (Armenian Hamster IgG) antibody was added in medium before incubation during the T-cell proliferation assay.

### RT-PCR and real-time RT-PCR

Total RNA was extracted from 10^6^ sorted thymocytes from C57BL/6 mice, using TRIzol (Invitrogen). Chloroform (0.2 ml; Sigma-Aldrich, USA) was added for every 1 ml of TRIzol used. Extracted RNA was shaken vigorously for 15 sec and incubated at room temperature for 2 min. After transferring the aqueous phase to a clean tube, isopropanol (Sigma-Aldrich) was added and the RNA was incubated at room temperature for 5 min. The RNA pellet was then washed with 1 ml of 75% ethanol, air-dried for 10 min, dissolved in 20 μl of water, and incubated at 55 °C for 10 min. The OD ratio (λ 260 nm/280 nm) was between 1.9 and 2.0, and RNA samples were assessed with electrophoresis on 1.5% agarose gels and visualized under UV light after ethidium bromide staining. RNA preparations were treated with DNase I to remove genomic DNA. cDNA was synthesized by incubating 20 μl of mRNA in a sprint C1000 terminal cycler (Bio-Rad, USA). Negative controls contained the reaction mixture except for template DNA. For quantification, relative mRNA expression of specific genes was obtained by the 2^−ΔΔCt^ method. The gene-specific primers (5′ → 3′) used are presented in Table 1. Data were normalized using transcripts for β-actin as controls, which were amplified with primers as presented in Table 1. Diluted cDNA (10 μl) was mixed with 2 μl of primer and 10 μl IQ SYBR Green SuperMix and was assayed in triplicate using a Stepone real-time system (ABI) under the following conditions: denaturation at 95 °C for 3 min, 40 cycles of 95 °C for 15 sec, and 60 °C for 1 min, followed by 30 sec of extension at 72 °C. Each sample was analyzed in triplicate.

**Table 1:** Primers used for RT-PCR to detect CTLA-4, Nanog mRNAs

β-actin sense	GAA ATC GTG CGT GAC ATC AAA G
Antisense	TGT AGT TTC ATG GAT GCC ACA G
CTLA-4 sense	TCTGCAAGGTGGAACTCA
Antisense	GCTAACTG CGACAAGGAT
Nanog sense	ATCCCGAGAACTATTCTTG
Antisense	GGTACTTCTGCTTCTGAAAC

### Statistical analysis

Mean and SD values were calculated with Microsoft Excel. At least three independent experiments were performed. A Tukey–Kramer post test was used to compare three or more means and a two-tailed unpaired Student’s *t* test was used to compare two groups (*p* ≤ 0.05 was considered statistically significant).

## Results

### CD117 and CD44 expression in different thymocyte subpopulations

**Fig. 1:**
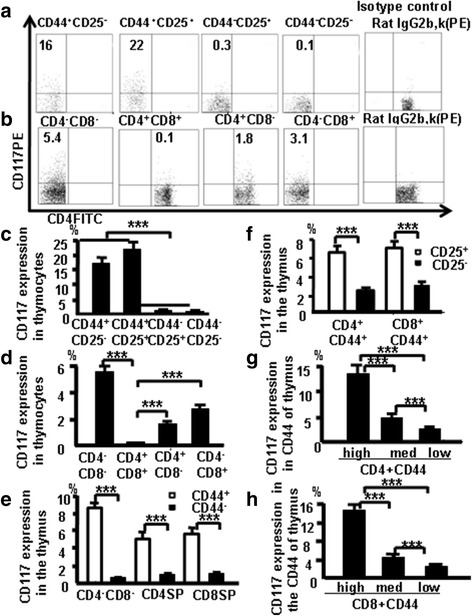
CD117 and CD44 expression in different thymocyte subpopulations. **a** CD117 expression in DN_1_, DN_2_, DN_3_, and DN_4_ thymocytes. **b** CD117 expression in DN, DP, and SP thymocytes. DN thymocyte data (**c**) and thymocyte data (**d**) pooled from three independent experiments. **e** CD44 expression analyzed in the different subpopulations of mouse thymocytes. **f** CD117 expression evaluated on CD4^+^CD44^+^CD25^+^ and CD8^+^CD44^+^CD25^+^ cells. **g** CD117 expression in CD44^+^CD4 SP cells, according to varying CD44 expression. **h** CD117 in CD44^+^CD8 SP cells, according to varying CD44 expression. Data are mean percent of total cells ± SD from three independent experiments. ***p < 0.001

CD117 plays an important role in the differentiation of T cells [9, 10]. T cells develop from CD3^–^CD4^–^CD8^–^c-kit^+^ cells through the modulation of cell surface molecules [27]. To confirm CD117 expression in the thymus during T-cell development, thymocytes were harvested from C57BL/6 mice, stained with CD4 (FITC), CD8 (PercP5.5), CD44 (APC-CY7), CD25 (PE-CY7), and CD117 (PE), and analyzed by flow cytometry. This group of antibodies is now termed the “antibody cocktail.” Isotype Rat IgG2b,k was used as a negative control (CD117). DN_2_ (CD44^+^CD25^+^) cells had the highest frequency CD117 expression, followed by DN_1_, DN_3_, and DN_4_ cells (Fig. 1a). Pooled data from three independent experiments are shown in Fig. 1c. DN cells had more CD117-expressing cells in C57BL/6 mice followed by CD8^+^ SP cells, CD4^+^ SP cells, and DP cells (Fig. 1b); the pooled data from three independent experiments (Fig. 1d) indicate that CD117 expression was lowest in DP cells.

Thymocytes from naive mice were stained with antibody cocktail to confirm whether CD117 expression is associated with CD44 expression in the other thymocyte subpopulations. In three experiments performed independently, more DN, CD4, and CD8 CD44^+^ cells expressed CD117 compared with DN, CD4 and CD8 CD44^–^ cells (Fig. 1e). There was also a positive correlation between CD25 and CD117 expression among CD4^+^CD44^+^ cells and CD8^+^CD44^+^ cells (Fig. 1f). Data (Fig. 1g, h) indicate that CD117 expressed in early stage T cells in the thymus positively correlates with CD44 in thymocytes.

### CTLA-4 expression correlates with CD25 and CD44 expression in different thymocyte populations

**Fig. 2:**
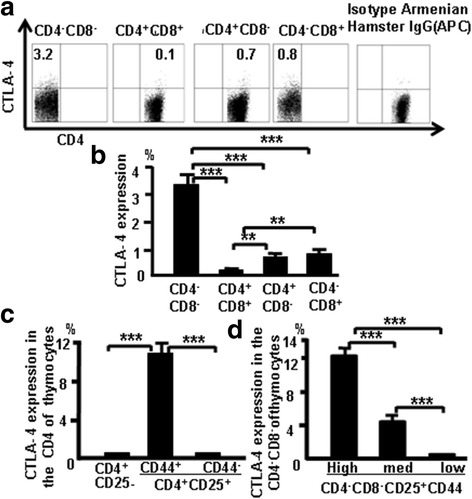
CTLA-4 expression correlates with CD25 and CD44 expression in different populations of thymocytes. **a** CTLA-4 expression in thymocytes of C57BL/6 mice. **b** CTLA-4 expression data pooled from three independent experiments. **c** CTLA-4 expression evaluated in CD4^+^CD25^–^ and CD4^+^CD25^+^ cells. **d** CTLA-4 expression in CD25^+^ DN cells, according to varying CD44 expression. Data are mean percent of total cells ± SD from three independent experiments. **p < 0.01, ***p < 0.001

CTLA-4 expression was examined in different subsets of thymocytes at various stages of T-cell development to confirm CTLA-4 expression in T-cell development. More DN cells expressed CTLA-4 compared with CD4 SP, CD8 SP, and DP cells (Fig. 2a). The pooled data from three independent experiments are shown in Fig. 2b, and the data show that CTLA-4 expression is greater in early stages of T-cell development. More CD4^+^CD25^+^CD44^+^ cells produced CTLA-4 compared with CD4^+^CD25^+^CD44^–^ and CD4^+^CD25^–^ cells (Fig. 2c, d). Data show that surface expression of CD25 and CD44 appears to be positively correlated with CTLA-4 expression.

### CTLA-4 correlates with Foxp3 expression in thymocytes

**Fig. 3:**
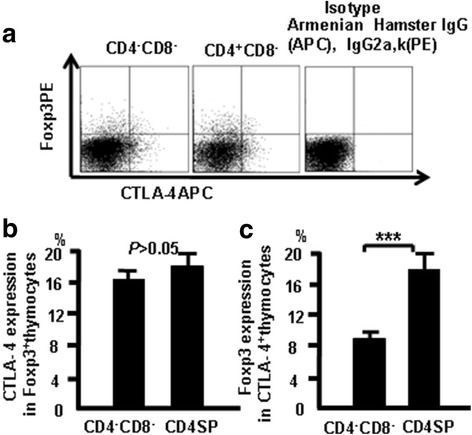
CTLA-4 expression correlates with Foxp3 expression in thymocytes. Cells were stained as in Fig. 1 with Foxp3 (PE) and analyzed by flow cytometry. **a** CTLA-4 and Foxp3 expression in different thymocyte populations. **b** CTLA-4 expression in Foxp3-positive thymocytes. **c** Foxp3 expression in CTLA-4-positive thymocytes. Data are mean percent of total and live thymocytes in each cell subset ± SD from three independent experiments. ***p < 0.001

Thymocytes from naive mice were stained with CD4, CD8, CD44, CD25, CTLA-4 (APC), and Foxp3 (PE), and were analyzed by flow cytometry to study the relationship between CTLA-4 and Foxp3. More CTLA4 expression was observed in Foxp3-positive CD4 SP cells compared with Foxp3-positive DN cells (Fig. 3). These data indicate that there is overlap of Foxp3 and CTLA-4 expression in some thymocytes.

### CTLA-4 expression in CD44^+^CD117^+^cells in the thymus

**Fig. 4:**
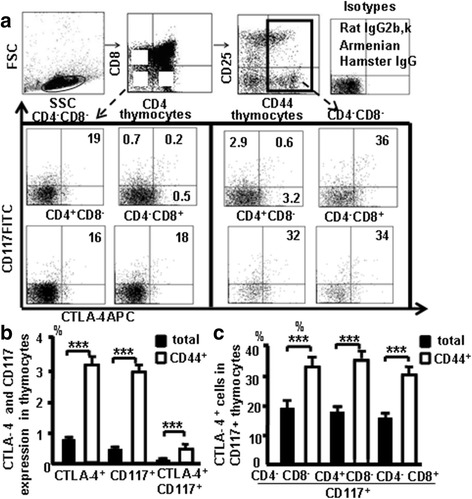
CTLA-4 expression in CD44+CD117+cells in the thymus. **a** CD117 and CTLA-4 expression in the different population in the thymocytes. **b** CD117 and CTLA-4 in thymocytes and in the CD44^+^ thymocytes. **c** CTLA-4 in CD117^+^ thymocytes and in CD117^+^CD44^+^ thymocytes. Data are mean percent of total ± SD from three independent experiments. ***p < 0.001

Thymocytes from naive mice were stained with the antibody cocktail and CTLA-4 (APC), we studied whether CD117 and CD44 expression correlates with CTLA-4 expression in the thymus (Fig. 4a). Data showed 0.5% of thymocytes were CD117^+^ cells, 0.7% of thymocytes were CTLA-4^+^ cells, and 0.2% of thymocytes were CTLA-4^+^CD117^+^ cells. Furthermore, 2.9% of the CD44^+^ thymocytes were CD117^+^ cells, 3.2% of the CD44^+^ thymocytes were CTLA-4^+^ cells, and 0.6% of the CD44^+^ thymocytes were CTLA-4^+^CD117^+^ cells (Fig. 4b). CTLA-4 expression was found in 19% of DN CD117^+^ cells, 18% of CD4^+^CD117^+^ cells, and 16% of CD8^+^CD117^+^ cells. Interestingly, CTLA-4 expression was also found in 34% of DN CD44^+^CD117^+^ cells, 36% of CD4^+^CD44^+^CD117^+^ cells, and 32% of CD8^+^CD44^+^CD117^+^ cells (Fig. 4c), demonstrating that CD44^+^ and CD117^+^ expression is closely associated with CTLA-4 in thymocyte stem T cells.

**Fig. 5:**
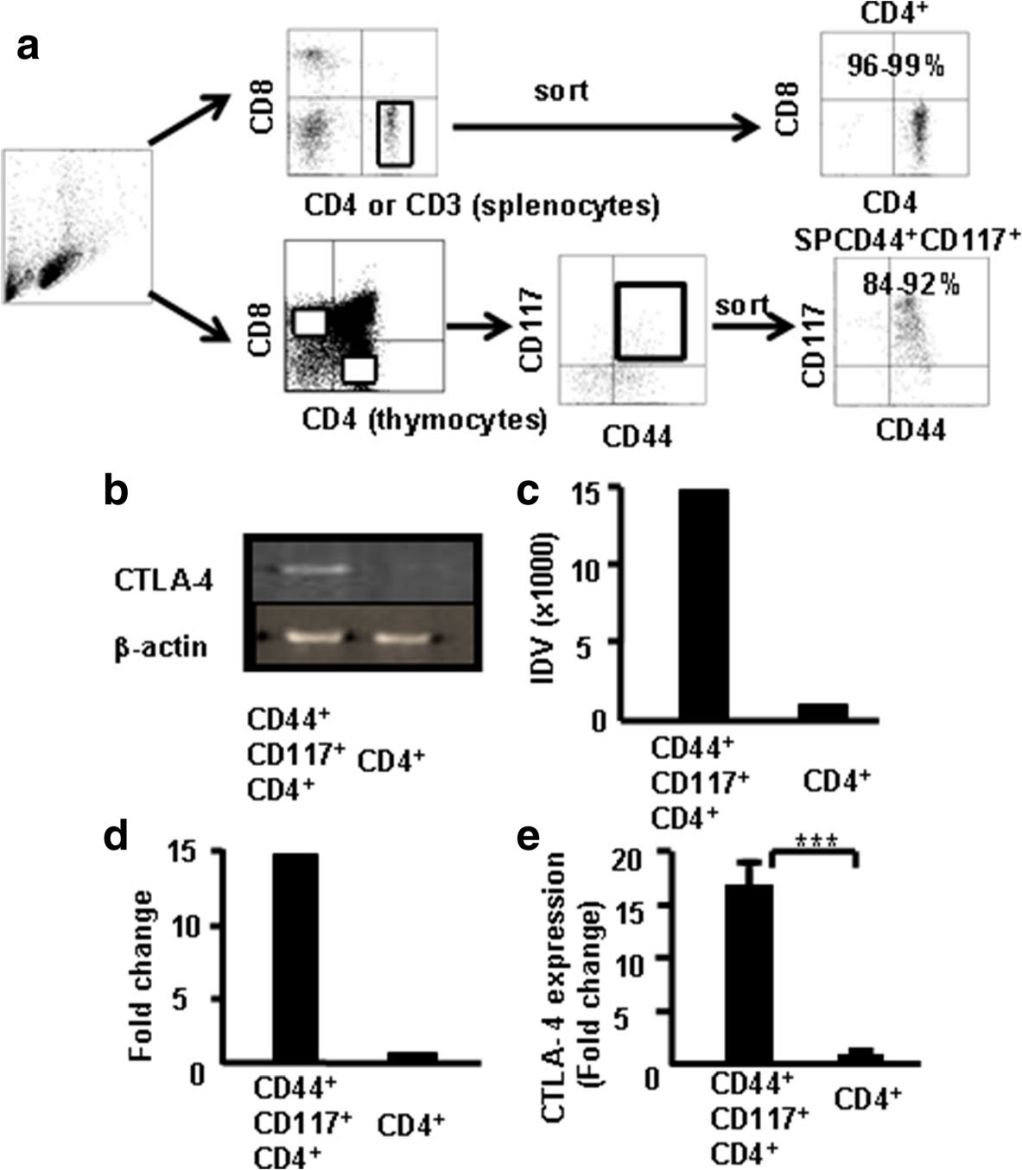
CTLA-4 expression in CD4^+^CD117^+^CD44^+^ thymocytes by RT-PCR and real-time RT-PCR. CD4^+^CD117^+^CD44^+^ and CD4^+^ SP cells sorted from the thymocytes of C57BL/6 mice (**a**). Total RNA extracted for RT-PCR and real-time RT-PCR analysis of CTLA-4 expression. **b** CTLA-4 expression in thymocytes by RT-PCR. **c** Integrated density value (IDV) for CTLA-4 transcripts quantified and normalized to β-actin. Representative results from one of three independent experiments. **d** Data pooled from three independent experiments. **e** Total RNA isolated for measuring CTLA-4 mRNA by real-time RT-PCR. Data are fold-induction relative to β-actin and representative results from one of three independent experiments. ***p < 0.001

CD4^+^ SP cells and CD4^+^CD44^+^CD117^+^ cells were sorted from the thymocytes via FACS (Fig. 5a) to further verify whether CD44^+^CD117^+^ cells express higher levels of CTLA-4. The expression of CTLA-4 mRNA in CD44^+^CD117^+^ cells and CD4^+^ SP cells was compared by RT-PCR. Results showed that the level of CTLA-4 was higher in CD4^+^CD44^+^CD117^+^ cells, as determined by densitometry measurements of the gels (Fig. 5b). Data from three independent experiments were pooled (Fig. 5c, d). Furthermore, CD4^+^ SP and CD4^+^CD44^+^CD117^+^ cells were sorted from the thymocytes and the levels of CTLA-4 mRNA were measured by real-time RT-PCR to further confirm the aforementioned findings. As shown in Fig. 5e, CD4^+^CD44^+^CD117^+^ cells expressed higher levels of CTLA-4 than CD4^+^ SP cells (~14× higher). These results corroborate the finding that CTLA-4 expression was much higher in CD4^+^CD44^+^CD117^+^ cells.

### CTLA-4 expression correlates with CD44 expression in different populations of CD117-positive thymocytes

**Fig. 6:**
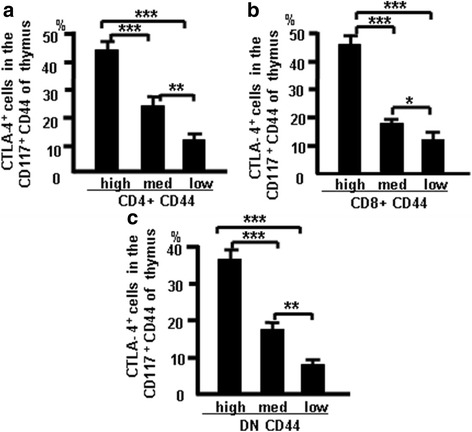
CTLA-4 correlates with CD44 expression in dif﻿fer﻿ent populations ﻿of CD117-positive thymocytes. Thymocytes were treated as indicated in Methods and analyzed by flow cytometry. **a** CTLA-4 expression in CD4^+^CD117^+^CD44^+^ cells. **b** CTLA-4 expression in CD8^+^CD117^+^CD44^+^ cells. **c** CTLA-4 expression in DNCD117^+^CD44^+^ cells. Data show mean percent of total cells ± SD from three independent experiments. *p < 0.05, **p < 0.01, ***p < 0.001. *DN* double-negative

Thymocytes were stained with the combination of antibodies to CD4, CD8, CD44, CD25, CTLA-4 (APC), and CD117 (PE) and analyzed by flow cytometry, and data show that CTLA-4 expression correlates with thymic CD44 expression. Fig. 6a–c show these data which suggest that CD44 expression positively correlates with CTLA-4 expression in CD117-positive thymocytes.

**Fig. 7:**
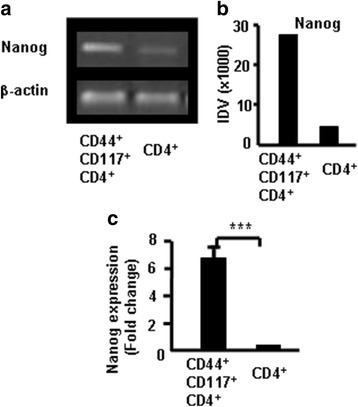
Nanog expression in CD117^+^CD44^+^ cells measured with RT-PCR and real-time RT-PCR. **a** Nanog in the thymocytes by RT-PCR. **b** Integrated density value (IDV) for the Nanog transcripts quantified and normalized to those of β-actin. **c** Total RNA isolated for measuring Nanog mRNA using real-time RT-PCR. Data presented as fold-induction relative to β-actin. Data represent one of three independent experiments. ***p < 0.001

CD44^+^CD117^+^ cells expressed more Nanog in thymocytes. Nanog is an important transcription factor for maintaining the self-renewal and pluripotency of ESCs. It regulates the fate of the inner cell mass of blastocysts in the embryo during early development. The homeoprotein Nanog is required for maintenance of pluripotency in mouse epiblast and ES cells [19]. To characterize the CD44^+^CD117^+^ cells in the thymus, CD4^+^ SP cells and CD4^+^CD44^+^CD117^+^ cells were sorted from the thymocytes via FACS to verify whether CD44^+^CD117^+^ cells express more Nanog. The Nanog mRNA expression in CD44^+^CD117^+^ cells and CD4^+^ SP cells was compared using RT-PCR. Results showed that Nanog were greater in CD4^+^CD44^+^CD117^+^ cells than in CD4^+^ SP cells as determined by densitometric analysis of gels (Fig. 7a). Pooled data from three independent experiments are shown in Fig. 7b. CD4 SP and CD4^+^CD44^+^CD117^+^ cells were sorted from thymocytes and Nanog mRNA expression was measured using real-time RT-PCR to confirm previous findings. CD4^+^CD44^+^CD117^+^ cells expressed more Nanog than CD4^+^ SP cells as shown in Fig. 7c. Data suggest that CD44^+^CD117^+^ cells are stem cells.

### CD44^+^CD117^+^ stem T cells from the thymus and spleen suppressed T-cell proliferation

**Fig. 8:**
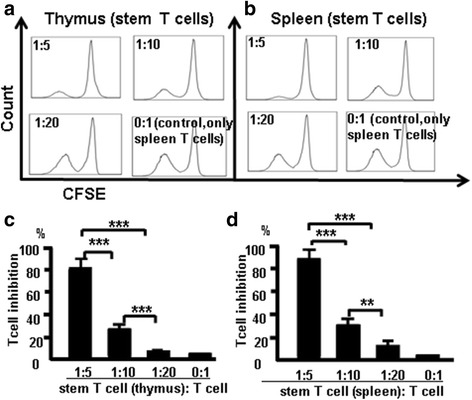
CD4^+^CD117^+^ cells from the thymus and spleen suppress T cells in vitro. T-cell proliferation was measured by flow cytometry to measure fluorescence intensity. **a** CD4^+^CD117^+^ cells sorted from the thymus. **b** CD4^+^CD117^+^ cells sorted from the spleen. Thymocyte data (**c**) and spleen data (**d**) pooled from three independent experiments. Data are mean percent of the total ± SD from three independent experiments. **p < 0.01, ***p < 0.001

CD117^+^ cells develop in the thymus and do not downregulate CD44 and CD117 expression, which is measurable in SP cells. We hypothesized that CD44^+^CD117^+^ T cells are stem T cells, which are key to the development and function of the immune system, especially of T cells. To confirm whether CD44^+^CD117^+^ T cells affect T-cell proliferation, stem T-cell function was examined in vitro using a CFSE assay. CD4^+^CD117^+^CD44^+^ T cells from the thymus were sorted by FACSAria II and were cocultured with T cells (CD4^+^CD117^+^CD44^+^ T cells:CD4^+^ T cells) in the presence of Con A (3 μg/ml). T-cell proliferation was measured by flow cytometry to determine the CFSE (5 μM) fluorescence intensity, as shown in Fig. 8. Stem T cells from the thymus and T cells from the spleen were sorted by FACSAria and were incubated with cocultured T cells (stem T cells:T cells) at ratios of 1:20, 1:10, and 1:5 in the presence of Con A. T-cell proliferation was measured by flow cytometry to determine the CFSE fluorescence intensity (Fig. 8a, b). The percentage of T-cell inhibition was 80%, 21%, and 5.1%, respectively. Further, both stem T cells and T cells from the spleen were sorted by FACSAria and were incubated with cocultured T cells (stem T cells:T cells) at ratios of 1:20, 1:10, and 1:5 in the presence of Con A. The percentage of cell inhibition was 84%, 26%, and 11%, respectively. The data from three independent experiments were pooled and are shown in Fig. 8c (stem cells from the thymus) and Fig. 8d (stem cells from the spleen). These results suggest that CD44^+^CD117^+^ T cells significantly suppressed T-cell proliferation.

### CTLA-4 suppressed T-cell proliferation and blocking CTLA-4 led to antibody-induced T-cell proliferation

**Fig. 9:**
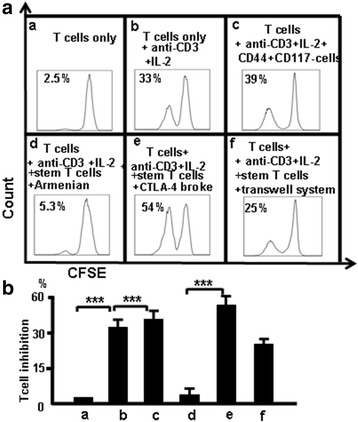
Blocking CTLA-4 reduces the ability of CD4^+^CD117^+^CD44^+^ cells to suppress T-cell proliferation. **a** *a* T cells only; *b* T cells + anti-CD3 + IL-2; *c* CD44^+^CD117^–^ T cells + anti-CD3 + IL-2; *d* CD44^+^CD117^+^ T cells + anti-CD3 + IL-2 + Isotype Armenian Hamster IgG; *e* (CD44^+^CD117^+^ T cells + CTLA-4 broke) + anti-CD3 + IL-2 + T cells; *f* CD44^+^CD117^+^ T cells cultured with T cells in a Transwell plate. **B** Data pooled from three independent experiments representing mean percent of totals ± SD. ***p < 0.001

CD44^+^CD117^+^ stem T cells from the thymus were sorted by FACSAria and incubated alone or cocultured (stem T cell:T cell) at a 1:2 ratio in the presence of anti-CD3 and IL-2 (300 u/ml) or blocking antibodies (1 μg/100 μl) to CTLA-4. Isotype Armenian Hamster IgG was used as a control. T-cell proliferation was measured by flow cytometry to determine the CFSE (2 μM) fluorescence intensity after 7-day incubation. The percentage of T-cell inhibition was 2.5% in T cells only (unstimulated) (Fig. 9Aa), 33% in T cells containing anti-CD3 + IL-2 (T cells only) (Fig. 9Ab), 5.3% in Isotype Armenian Hamster IgG (control, T cells were cultured with CD44^+^CD117^+^ T cells and Isotype Armenian Hamster IgG together) (Fig. 9Ac), 39% in T cells + CD44^+^CD117^–^ T cells containing anti-CD3 + IL-2 (control, T cells were cultured with CD44^+^CD117^–^ T cells) (Fig. 9Ad), 54% in the medium containing CTLA-4 broke antibody (CD44^+^CD117^+^ T cells were cultured with CTLA-4 broke, after 1 h the cells were washed and cultured with T cells in the medium) (Fig. 9Ae), and 25% in CD44^+^CD117^+^ T cells and T cells in Transwell plate coculture system (CD44^+^CD117^+^ T cells and T cells were cultured in the Transwell system) (Fig. 9Af). The data from three independent experiments were pooled and are shown in Fig. 9B. Data show that CD44^+^CD117^+^ stem T cells suppressed T-cell proliferation by the interaction between the cells, and suppressive activity of stem T cells was reduced in the presence of anti-CTLA-4 antibody.

### CD44^+^CD117^+^ stem T cells can self-renew

**Fig. 10:**
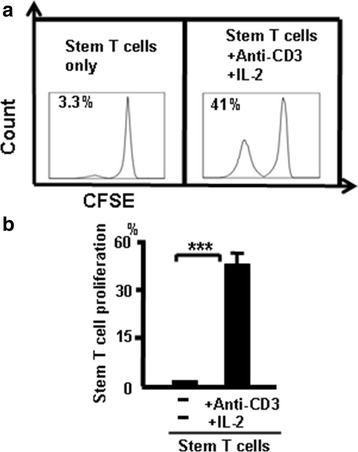
CD44^+^CD117^+^ T cells have the ability to self renew. **a** CD4^+^CD44^+^CD117^+^ T cells only and CD4^+^CD44^+^CD117^+^ T cells + anti-CD3 + IL-2. **b** Data pooled from three independent experiments representing mean percent ± SD. ***p < 0.001

To confirm that CD44^+^CD117^+^ T cells can self-renew, thymic CD44^+^CD117^+^ stem T cells were sorted with FACSAria and incubated alone or cocultured (stem T cell:T cell) at a 1:2 ratio in the presence of anti-CD3 and IL-2 (300 u/ml). T-cell proliferation was measured by flow cytometry to measure CFSE (2 μM) fluorescence intensity after 7-day incubation. T-cell proliferation was low in CD44^+^CD117^+^ stem T cells only (unstimulated), and this was greater in CD44^+^CD117^+^ T cells containing anti-CD3 + IL-2 (Fig. 10). Thus, CD44^+^CD117^+^ T cells can self-renew.

### CD44^+^CD117^+^ cells in blood and lymph nodes

**Fig. 11:**
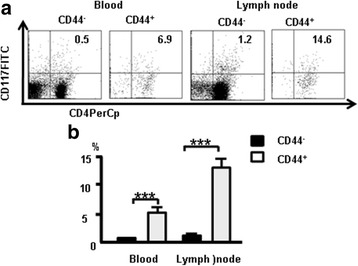
CD44^+^CD117^+^ cells in blood and lymph nodes. Cells from naive mice were stained with the combination of antibodies CD4 (FITC), CD8 (PerCp), CD25 (PE-CY7), CD44 (APC-CY7), and CD117 (FITC) and analyzed by flow cytometry. **a** CD117 expression and CD44^+^CD117^+^ cells in blood and lymph nodes. **b** Data pooled from three independent experiments and represent mean percent ± SD. ***p < 0.001

To confirm CD44^+^CD117^+^ cells ar﻿e ﻿exist in blood and lymph nodes, cells from naive mice were stained with the combination of antibodies CD4 (FITC), CD8 (PerCp), CD25(PE-CY7), CD44 (APC-CY7), and CD117 (FITC) and analyzed by flow cytometry. The results showed 0.5% of CD4^+^CD117^+^ cells and 1.2% of CD4^+^CD117^+^ cells in blood and lymph nodes, respectively (Fig. 11). Furthermore, 6.9% of CD4^+^ CD117^+^ cells and 14.6% of CD4^+^ CD117^+^ cells were present in CD44^+^ cells of blood and lymph nodes, respectively. These results suggest CD44^+^CD117^+^ T cells are ﻿exist in blood and lymph nodes.

**Fig. 12:**
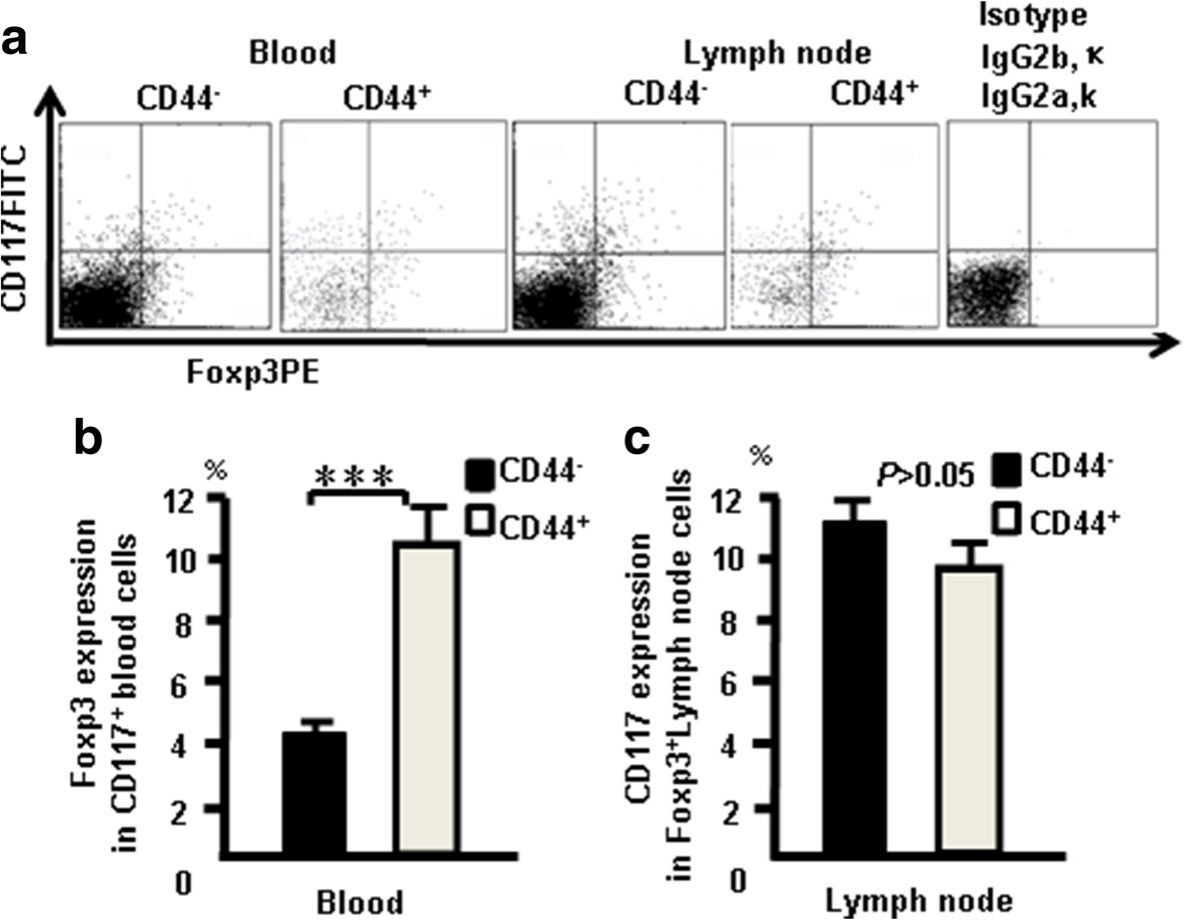
CD44^+^CD117^+^ cells expressed Foxp3 in blood and lymph nodes. Cells from naive mice were stained with the following combination of antibodies: the antibody cocktail with CD117 (FITC) and Foxp3 (PE). **a** CD117 and Foxp3 expression in blood and lymph nodes. **b** Foxp3 expression in CD117^+^ blood cells. **c** Foxp3 expression in CD117^+^ lymph node cells. Data pooled from three independent experiments and represent mean percent ± SD. ***p < 0.001

CD44^+^CD117^+^ cells expressed Foxp3 in blood and lymph nodes. The cells from naive mice were stained with the following combination of antibodies: the antibody cocktail with Foxp3 (PE) and used to confirm whether CD117 and CD44 expression correlates with Foxp3 expression in the blood and lymph nodes. Data show that 4.5% of CD4^+^CD117^+^ cells expressed Foxp3, 10.3% of CD4^+^CD44CD117^+^ cells expressed Foxp3^+^ in blood cells, 11% of CD4^+^Foxp3^+^ cells expressed CD117, and 9.5% of CD4^+^CD44^+^ cells expressed Foxp3 in lymph nodes (Fig. 12a). The pooled data from three independent experiments are shown in Fig. 12b, c. These results suggested that CD44^+^CD117^+^ cells and nT_reg_ cells are two different populations of cells, although part of them overlap.

## Discussion

Using an advanced flow cytometry method, we  researched the characteristic of  T-cell development in the thymus by measuring expression of CD117 and CTLA-4 in thymocytes. Surprisingly, CD117^+^ stem T cells were found in mature thymocytes and CD117 and CTLA-4 expression occurred at each stage of T-cell development. Changes in CD25, CD44, CD117, and CTLA-4 expression were examined in DN, DP, and SP cells using multi-color flow cytometry. CD117 was expressed in the early stages (DN_1_ and DN_2_) of T cells (Fig. 1a, c), and DN cells expressed more CD117 than did DP and SP cells (Fig. 1b, d). CD117 expression was positively correlated with CD44 in thymocytes (Fig. 1e–h). CD44^+^CD117^+^ T cells could be also found in blood and lymph nodes (Fig. 11). CD4SP and CD8SP cells contain many CD117^+^ cells, most of which are CD44^+^CD117^+^ cells. CD44^+^CD117^+^ cells have been used to label tumor stem cells [8, 10, 23, 28]. In the current study, CD117 and CD44 were used to label stem T cells and development stages were identified in the thymus.

Next, CTLA-4 expression was measured at different stages of T-cell development. CTLA-4 expression was higher in DN cells compared with SP and DP cells (Fig. 2a, b) and was positively correlated with CD25 and CD44 (Fig. 2c, d). CTLA-4 and Foxp3 overlapped in DN and CD4^+^cells (Fig. 3). CD44^+^CD117^+^ cells and nT_reg_ cells are two different populations of cells, although parts of them overlap (Fig. 12). CD44^+^CD117^+^ cells had more CTLA-4 expression (Figs. 4 and 5). CD44 expression was positively correlated with CTLA-4 in CD117^+^ thymocytes (Fig. 6). CD117^+^CD44^+^ T cells are stem T cells that expressed more Nanog than other T cells in thymocytes (Fig. 7) and have the ability to self-renew (Fig. 10), confirming that CD44^+^CD117^+^ stem T cells suppressed T-cell proliferation and CD4^+^CD117^+^CD44^+^ thymus cells had a potent suppressive function (Fig. 8). Because there is more CTLA-4 expression in stem T cells, we studied the effect of CTLA-4 on stem T cells. CD117^+^ stem T cells were incubated with CTLA-4 blocking antibodies to confirm whether blocking CTLA-4 abrogated the suppressive mechanism of CD117^+^ stem T cells. CD44^+^CD117^+^ stem T cells and T cells proliferated, and when cultured separately this proliferation ceased. Blocking CTLA-4 abrogated the suppressive function of CD44^+^CD117^+^ stem T cells, and induced T-cell proliferation (Fig. 9). The suppressive activity of stem T cells was partly aborted by anti-CTLA-4 antibodies and the ability of CD44^+^CD117^+^ cells to inhibit T cells was diminished in a Transwell plate, suggesting that this inhibition depends on cell-to-cell contact.

Stem T cells in the immune system differ from other stem cells with respect to development and why this is true is unclear. Research for study the effects of MSCs on lymphocyte proliferation and their immune modulation has provided invaluable data [29]. Stem T cells are analogous to MSCs; however, they highly express abundant CTLA-4 and have a greater ability to modulate the immune system. How stem T cells interact with, and regulate, T cells, B cells, and natural killer cells, DCs, or neutrophils is unclear, as is whether stem T cells alter lymphocyte phenotype or induce development of T regulatory cells in vitro and/or in vivo, or whether stem T cells are anti-inflammatory in vivo*.* Tumor cells are associated with tumor stem cells [30], but CD44^+^CD117^+^ stem T cells regulate T-cell proliferation. The difference between tumor stem cells and CD44^+^CD117^+^ stem T cells is worthy of exploration.

**Fig. 13:**
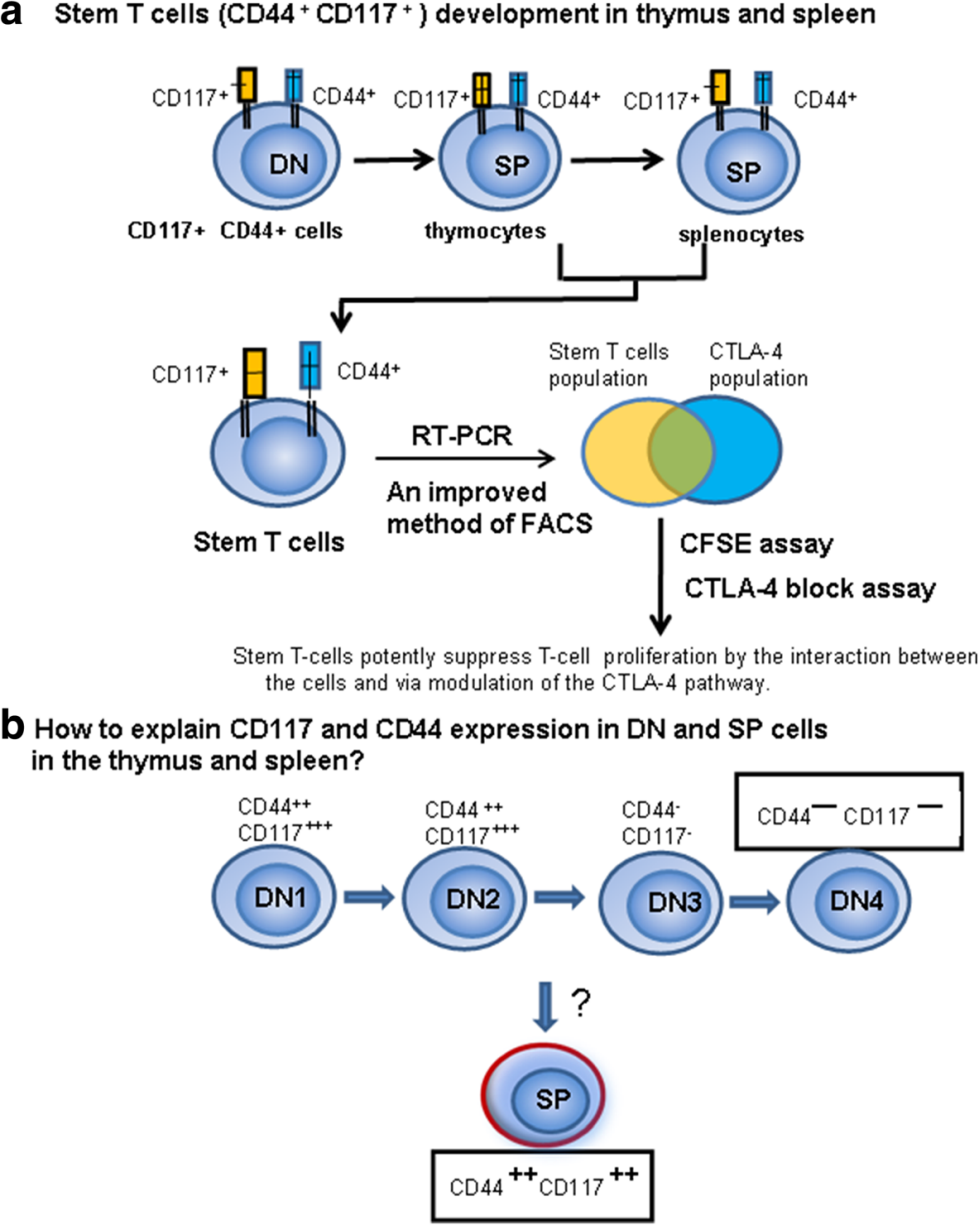
Stem T-cell development in the thymus and spleen. **a** Methods of stem T-cell development. **b** Important question of whether there is a contradictory problem with traditional DN T-cell development theory. *DN* double-negative, *SP* single-positive

CD44^+^CD117^+^ stem T-cell development in the thymus and spleen. We sought to identify various gene/protein expression changes at different stages of T-cell development. Many changes were identified, which are contradictory to the current theory of T-cell development. In this study, peak CD117 expression occurred in DN and CD117 cells and could still be detected in SP T cells in the thymus and spleen (Fig. 13). Thus, studies are needed to explain this phenomenon.

Previous work indicates that CD117 cells are present in DN_1_ and DN_2_, but not in SP cells [31]. We observed that CD117 cells such as NK T cells and T_regs_ develop in the thymus, and do not follow the theory of T-cell or DN T-cell development. CD117 cell development may begin from CD44^+^ DN cells and proceed directly to CD44^+^ SP cells. It is unclear why NK T cells, T_regs_ and CD44^+^CD117^+^ cells are T cells in phenotype but do not share the T-cell development pathway. We propose that CD44^+^CD117^+^ T cells may not have been derived from a common precursor as T cells, but rather pass through a different pathway.

## Conclusions

In summary, CD44^+^CD117^+^ T-cell development occurred in the thymus and spleen. CD44^+^CD117^+^ T cells expressed abundant CTLA-4 and suppressed T-cell proliferation. Blocking CTLA-4 reduced the suppression of T-cell proliferation. CD44^+^CD117^+^ T cells are stem cells that expressed more Nanog and CTLA-4 and potently suppressed T-cell proliferation via modulating the CTLA-4 pathway. Finally, CD44^+^CD117^+^ T cells may have developed from CD4^–^CD8^–^CD44^+^CD25^–^ T progenitor cells, without the involvement of DN_3_, DN_4_, and CD4^+^CD8^+^ (DP) stages in the thymus.

## Abbreviations

CTLA-4: Cytotoxic T lymphocyte antigen-4
DN: Double-negative CD4^–^CD8^–^
DN_1_: Double-negative CD44^+^CD25^–^
DN_2_: Double-negative CD44^+^CD25^+^
DN_3_: Double-negative CD44^–^CD25^+^
DN_4_: Double-negative CD44^–^CD25^–^
DP: Double-positive CD4^+^CD8^+^
SP: Single-positive CD4 or CD8
